# Genetic Patterns of Paternity and Testes Size in Mammals

**DOI:** 10.1371/journal.pone.0009581

**Published:** 2010-03-08

**Authors:** Carl D. Soulsbury

**Affiliations:** School of Biological Science, University of Bristol, Bristol, United Kingdom; University of Arizona, United States of America

## Abstract

**Background:**

Testes size is used as a proxy of male intrasexual competition, with larger testes indicative of greater competition. It has been shown that in some taxa, social mating systems reflect variance in testes size, but results are not consistent, and instead it has been suggested that genetic patterns of mating may reflect testes size. However, there are different measures of genetic patterns of mating. Multiple paternity rates are the most widely used measure but are limited to species that produce multi-offspring litters, so, at least for group living species, other measures such as loss of paternity to males outside the social group (extra group paternity) or the proportion of offspring sired by the dominant male (alpha paternity) might be appropriate. This study examines the relationship between testes size and three genetic patterns of mating: multiple paternity, extragroup paternity and alpha paternity.

**Methodology/Principal Findings:**

Using data from mammals, phylogenetically corrected general linear models demonstrate that both multiple paternity and alpha paternity, but not extra group paternity, relate to testes size. Testes size is greater in species with high multiple paternity rates, whereas the converse is found for alpha paternity. Additionally, length of mating season, ovulation mode and litter size significantly influenced testes size in one model.

**Conclusions/Significance:**

These results demonstrate that patterns of mating (multiple paternity and alpha paternity rates) determined by genetic analysis can provide reliable indicators of male postcopulatory intrasexual competition (testes size), and that other variables (length of mating season, ovulation mode, litter size) may also be important.

## Introduction

Polyandry, i.e. multiple-mating by females, is known to be widespread across the animal kingdom [1], [2]. Genetic evidence indicating that offspring within litters or clutches can be sired by different males has been found in invertebrates, fish, amphibians, reptiles, birds and mammals [2]–[7]. A consequence of female multiple-mating is that ejaculates from different males may overlap in the female's reproductive tract and compete to fertilize her ova (sperm competition; [8]). Increasing the number of sperm inseminated may increase the competitive advantage of one male over another. However, ejaculates are costly to produce [9], and male investment in the number of sperm should reflect species-specific and locality-specific variance in the degree or likelihood of sperm competition [9], [10]. As the demand for more sperm increases, so the investment in testicular tissue increases [11]–[13].

Variation in post-copulatory male intrasexual competition alters testes size. In particular, in mating systems where male post-copulatory intrasexual competition is high, relative testes size (testes mass controlled for body mass) is generally high [14]. However, evidence that the mating system observed from behaviour (social mating system) relates to relative testes size has been mixed in mammals and birds [15], [16], and studies have indicated that inferring sperm competition levels from social mating systems can be misleading [17], as these may differ greatly from mating system deduced from DNA analysis (genetic mating systems) [18]. Additionally, relative testes size is sensitive to other life history and ecological traits such as the length of the mating season and ovulation mode, as this may alter levels of male post-copulatory intrasexual competition [15], [19]. Given the disparity that can occur between social and genetic mating systems, it has been suggested that using data from genetic mating patterns may provide better quantitative measures of sperm competition levels [20]. There are however different ways of measuring genetic mating patterns; for example, the presence (but not absence) of multiple sires in a litter (multiple paternity) indicates the presence of sperm competition. In turn, rates of multiple paternity in a population may be indicative of level of male post-copulatory intrasexual competition levels. Multiple paternity cannot occur in species that produce single offspring (monotocous species) e.g. most primates. For some species that live in groups, the proportion of all offspring sired by the dominant male (alpha paternity) or the proportion sired by males outside the social group (extra group paternity) may provide a measure of male intrasexual competition. Multiple paternity, alpha paternity and extra group paternity provide distinct information on mating systems and strategies, but it is not clear how they may influence male post-copulatory intrasexual competition, and so whether they reflect variation in relative testes size. Though measures may be considered interchangeable [20], the fact that they represent distinct information about patterns of mating means suggest that they might not relate to male post-copulatory intrasexual competition in the same way. Therefore an *a priori* assumption that all measures correlate to relative testes size cannot be made.

So far studies looking at both inter- and intraspecific comparisons have found that relative testes size increases with multiple paternity rates [21]–[23]. However, such analyses have been limited to small samples sizes within the order Rodentia ([21]: *n* = 14 species; [22]: *n* = 4 species; [23]: *n* = 1 species, 7 populations), and it is unclear whether this pattern extends more widely across mammals. Additionally, no studies have examined extra group paternity or alpha paternity rates in relation to relative testes size across mammals. To bridge this gap, I report the frequency of multiple paternity, extra group paternity and alpha paternity for mammals and examine their relationship with relative testes size, in addition to length of mating season, litter size and ovulation mode, variables previously shown to be important predictors of relative testes size. I was able to show that relative testes size significantly relates to genetic measures of paternity and in one model, to other variables that may alter male post-copulatory intrasexual competition.

## Results

### Relative Testes Size and Multiple Paternity

**Figure 1:**
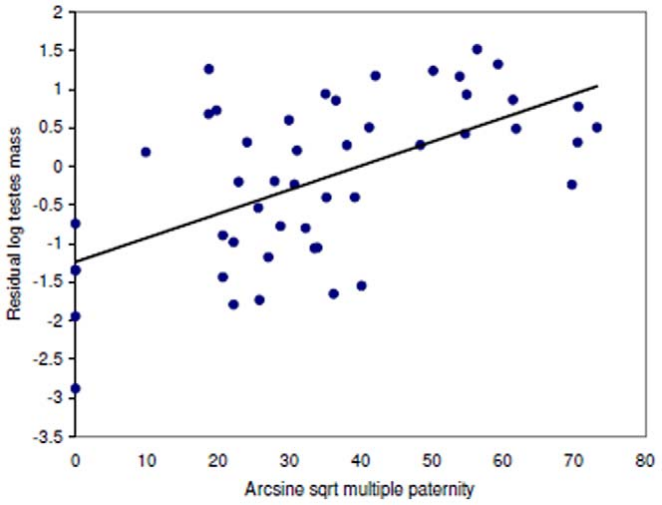
Relative testes size and multiple paternity rates. Regression line (*y* = 0.0311×- 1.2339) shown through the phylogenetically corrected residual testes size. Multiple paternity rate is significant in the full model (*t* = −2.90, *P* = 0.006; see Table 1).

**Figure 2:**
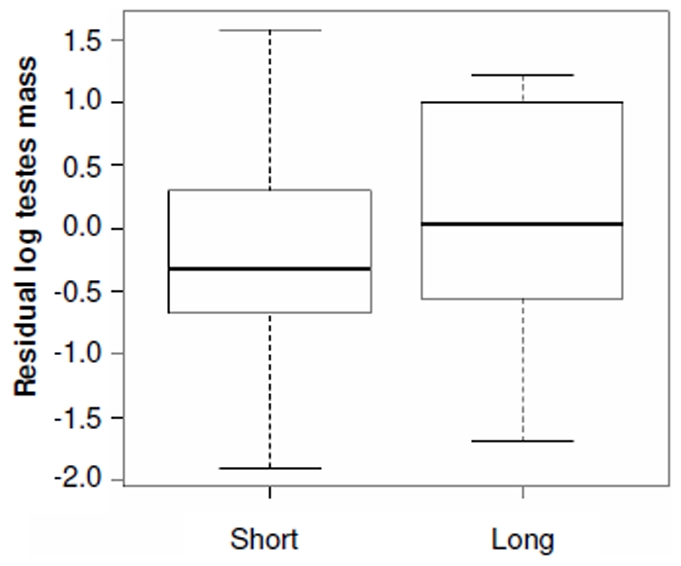
Relative testes size and length of mating season. Short mating season (<6 months); Long mating season (≥6 months). Length of mating season is significant in the full model (*t* = −2.90, *P* = 0.006; see Table 1).

**Figure 3:**
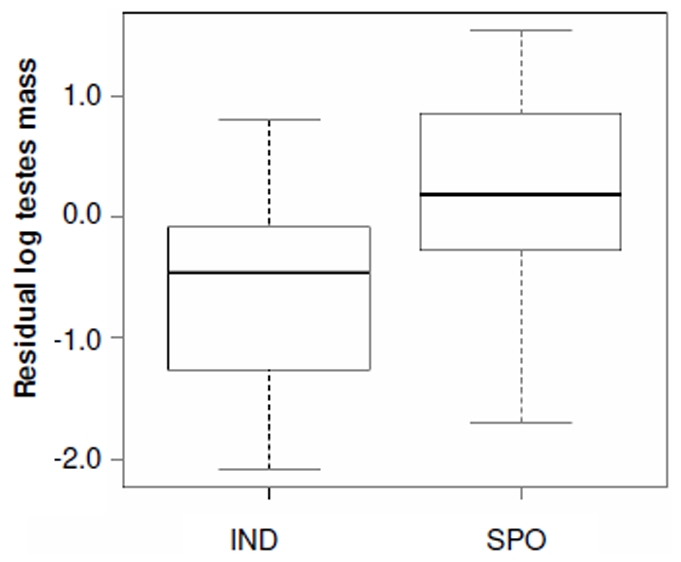
Relative testes size and ovulation mode. Ovulation mode is significant in the full model (*t* = 4.16, *P*<0.001; see Table 1).

**Figure 4:**
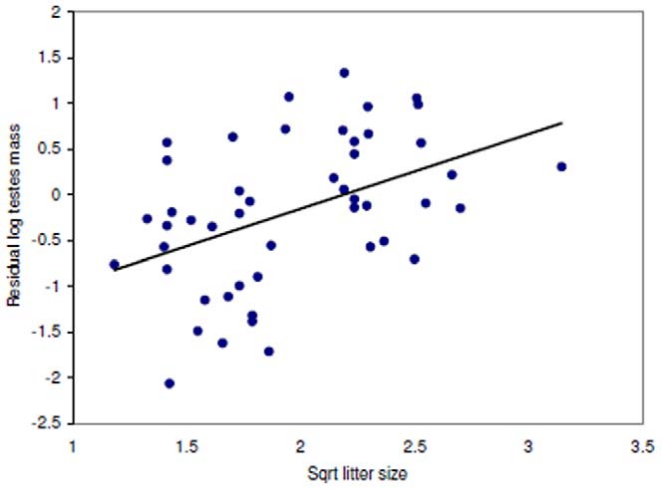
Relative testes size and litter size. Regression line (*y* = 0.8173×- 1.7911) shown through the phylogenetically corrected residual testes size. Litter size is significant in the full model (*t* = 3.32, *P* = 0.001; see Table 1).

Mean (±SE) multiple paternity was found in 35.6±2.8% of litters (range 0–92%; *n* = 67 species, 86 values, 9 mammalian orders). Testes and ovulation data were available for 49 species. Relative testes size was associated with multiple paternity, length of mating season and litter size, but not ovulation mode (Table 1). However, Grubb's test identified a single outlier (Z_0.05_ = 2.83); the removal of this species (spotted hyena *Crocuta crocuta*) did not appreciably alter the previous significant relationships (Table 1), but did make ovulation mode significant (Table 1). The spotted hyena is unusual in that females have significant control over mate choice [24]; this may be lowering male post-copulatory intrasexual competition and be the cause of this species as an outlier. Overall, relative testes size was positively correlated to multiple paternity rates (Figure 1) and was lower in species with short mating seasons (Figure 2). Spontaneous ovulators had higher relative testes size than induced ovulators (Figure 3), whilst relative testes size was positively correlated to litter size (Figure 4).

**Table 1:** Outputs of the PGLMs (slope, *t* & *P*) with effect sizes (*r*) and 95% confidence intervals (CI) for relative testes size (testes mass controlled for body mass) and models containing (i) multiple paternity (*R* ^2^ = 0.79, *F*_6,49_ = 38.58, *P*<0.001), (ii) multiple paternity without an outlier (*R* ^2^ = 0.83, *F*_6,48_ = 46.39, *P*<0.001), (iii) extra group paternity (*R* ^2^ = 0.86, *F*_5,13_ = 26.48, *P*<0.001) and (iv) alpha paternity (*R* ^2^ = 0.84, *F*_5,17_ = 22.36, *P*<0.001).

Dependent variable	MLλ	Predictor variables^a^	slope	*t*	*P*	*r* b	CI^c^
Testes mass	0.85^e^	Body mass	0.90	13.18	<0.001	0.82	0.82/0.92
		Multiple paternity	0.02	3.65	0.001	0.46	0.21/0.63
		Long MS	0				
		Short MS	−0.63	−2.51	0.016	−0.34	−0.54/−0.07
		Ovulation (IND)	0				
		Ovulation (SPO)	0.45	1.67	0.099	0.23	−0.05/0.46
		Litter size	0.97	3.55	0.001	0.45	0.20/0.63
Testes mass^d^	0.85^e^	Body mass	0.92	15.46	<0.001	0.91	0.86/0.94
		Multiple paternity	0.02	4.16	<0.001	0.51	0.28/0.67
		Long MS	0				
		Short MS	−0.69	−2.90	0.006	−0.39	−0.58/−0.12
		Ovulation (IND)	0				
		Ovulation (SPO)	0.654	2.50	0.016	0.34	0.07/0.55
		Litter size	0.86	3.32	0.001	0.43	0.18/0.61
Testes mass	0.00^f^	Body mass	0.89	6.62	<0.001	0.89	0.69/0.94
		EGP	0.01	0.56	0.583	0.16	−0.38/0.49
		Long MS	0				
		Short MS	0.33	0.47	0.645	0.13	−0.40/0.57
		Ovulation (IND)	0				
		Ovulation (SPO)	−0.19	−0.25	0.810	−0.07	−0.54/0.44
Testes mass	0.47e,f	Body mass	0.81	8.06	<0.001	0.88	0.75/0.94
		Alpha paternity	−0.03	−2.32	0.032	−0.48	−0.72/−0.04
		Long MS	0				
		Short MS	0.49	1.11	0.239	0.25	−0.21/0.59
		Ovulation (IND)	0				
		Ovulation (SPO)	−1.03	−1.71	0.105	−0.37	−0.66/0.08

a SPO: spontaneous ovulation, IND: induced ovulation; Long MS: long mating season, Short MS: short mating season.

b Conventions for effect sizes: small *r* = 0·10, medium *r* = 0·30, large *r* = 0·50 [47];

c relationships are significant where CI exclude zero;

d model without single outlier;

e significantly different from 0;

f significantly different from 1.

**Figure 5:**
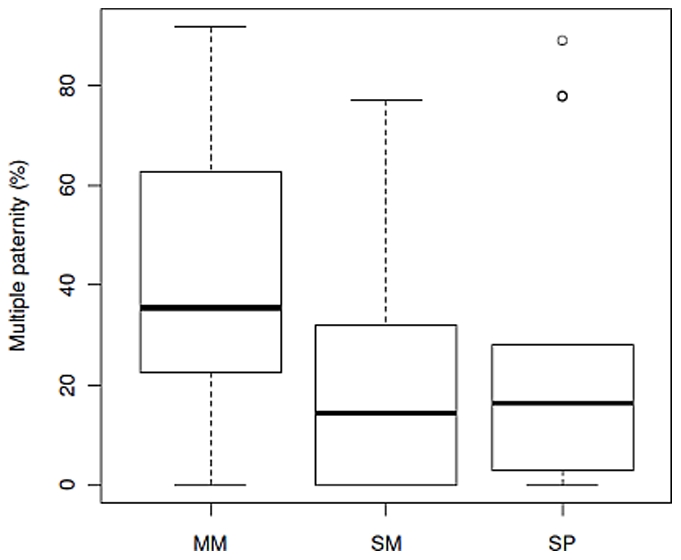
Multiple paternity rates and social mating system. SM: socially monogamous; SP: socially polygynous; MM: multi-male. Multiple paternity rates differed significantly between social mating systems (*F*_2,61_ = 4.58 *P* = 0.014); SM was significantly different from MM species (Tukey HSD = 0.036), but not SP species (Tukey HSD = 0.839). MM and SP species did not differ significantly (Tukey HSD = 0.079).

Multiple paternity rates differed significantly between social mating systems (*F*_2,61_ = 4.58 *P* = 0.014). Post-hoc testing indicated that socially monogamous (SM) species had a significantly lower frequency of multiple paternity than multi-male (MM) species (Tukey HSD = 0.036), but socially polygynous (SP) species did not differ between either category (vs. SM: Tukey HSD = 0.839; vs. MM: Tukey HSD = 0.079; Figure 5).

### Relative Testes Size, Extra Group Paternity and Alpha Paternity

Across eight mammalian orders, mean (±SE) extra group paternity was 21.6±3.6% of offspring (range 0–77%; *n* = 39 species, 40 values). Testes mass data were available for 17 species. Relative testes size was not related to any variable (Table 1), nor did extra group paternity differ among social mating systems (*F*_2,37_ = 0.55, *P* = 0.581).

**Figure 6:**
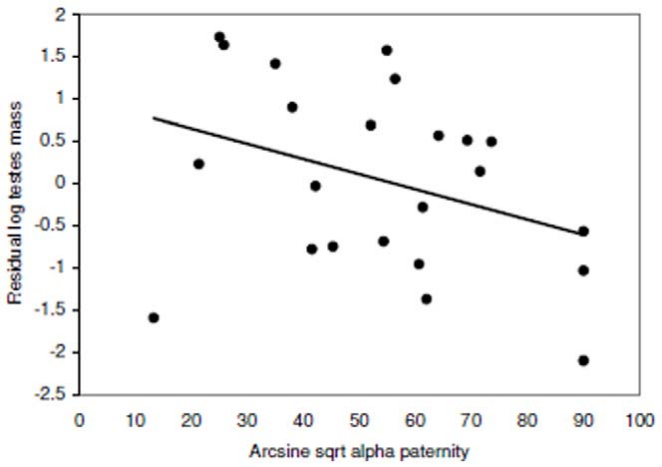
Relative testes size and alpha paternity. The regression line (*y* = 0.511*×*-0.492) shown through the phylogenetically corrected residual testes size. Alpha paternity is significant in the full model (*t* = −2.32, *P* = 0.032; see Table 1).

Mean (± SE) alpha paternity was 66.6±4.1% of offspring (range 5.3–100%; *n* = 41 species, 7 mammalian orders). From 21 species with testes and ovulation data, relative testes size was related to alpha paternity only (Table 1), with a significant negative relationship between relative testes size and alpha paternity (Figure 6). Alpha paternity did not vary significantly between mating systems (*F*_2,37_ = 0.718, *P* = 0.494).

## Discussion

### Relative Testes Size and Multiple Paternity

Multiple paternity was frequent in mammals (*x*– = 36% litters), but lower than rates found in reptiles (*x*– = ∼50% clutches: [7]), possibly because more reptiles mate promiscuously (80%: [25]). In contrast, 19% of socially monogamous bird broods (90% of bird species: [26]) contained extra-pair offspring [17], but it is unclear whether this reflects multi-sired or single paternity broods, though the extra pair male is unlikely to sire the entire brood (e.g. [3]). Thus rates in mammals appear to be higher than birds, probably reflecting a greater proportion of promiscuous mating systems than in birds, but lower than reptiles. Mammalian multiple paternity rates did not show any concordance with social mating systems, though multi-male species tended to have higher multiple paternity rate than other categories. Thus, social mating system was a poor indicator of male intrasexual competition as found in studies of single taxa (e.g. voles, [27]; carnivores, [15]), but this result may not be consistent in monotocous species which cannot show multiple paternity e.g. primates [28].

Higher levels of multiple paternity were associated with larger relative testes size, as was previously shown in rodents [21]. This confirms the hypothesis that relative testes size reflects the intensity of male post-copulatory intrasexual competition across multiple mammalian taxa. Such a relationship may be expected to be stronger if more testes data came from the same population as the genetics data, as studies have indicated that local variation in male post-copulatory intrasexual competition can alter testes size [23], [29]. In my dataset, small testes were sometimes found in species with high multiple paternity rates. This may reflect the limitations of using testes data from different localities, or that multiple paternity rates may vary temporally within a population (e.g. [30]). Thus, disparities between relative testes size and multiple paternity rate may reflect an evolutionary disequilibrium with behaviour evolving faster than morphological traits [31]. It has been shown experimentally that variation in male post-copulatory intrasexual competition can alter insect testes size [32]. However, it is not known whether variation in multiple paternity rates causes variation in testes size within a single population of mammals and, how fast testes size responds to any variation. Consequently, this represents an important aspect for future study.

In contrast to patterns observed in a single order [15], relative testes size was lower in short mating season species This contrast with the hypothesis that increased female reproductive synchrony promotes male intrasexual competition for receptive females and male investment in spermatogenic tissue [15], [19], [33]. However, the majority of species used in the analysis of a single order produce 1 litter/reproductive cycle. In this dataset, many species, particularly rodents, have the ability to produce multiple litters. As a consequence, the increased opportunity to sire litters over a long period would appear to be an important influence on relative testes size [e.g. 34].

Spontaneous ovulators had greater relative testes size than induced ovulators. As copulation triggers ovulation in induced ovulators, the male that successfully induces ovulation may sire a greater proportion of the offspring [35], whereas for spontaneous ovulators, the male copulating closest to ovulation generally sires most of her offspring [36]. However, males of spontaneous ovulators cannot predict the exact timing of ovulation so their sperm may be outcompeted by other males' ejaculates, leading to an increase in male-male competition. Across mammals, spontaneous ovulators have higher sperm concentrations and produce ejaculates with greater numbers of sperm [37]. Thus, it would be expected that the demand for more sperm is linked to larger testes size in spontaneous ovulators as found in this study. Taken together, these data indicate that across mammals, ovulation mode is a powerful driver of sperm competition levels and future cross-species comparative studies should include ovulation mode as a variable where both modes are present in a dataset.

Litter size was also positively related to relative testes size, something that has not been previously reported in mammals. Clutch sizes in birds have also been found to be positively correlated to testes size [38] and it has been suggested that this is caused by increased copulation frequency associated with larger clutch sizes and resulting sperm depletion [38]. However, copulation frequency and clutch size do not correlate [39] and these results may have been an artifact of the geographical spread of the data rather than an effect of sperm competition; hence, firm conclusions remain to be made in birds [38]. If fertilization success is determined by the ‘raffle principle’ (*sensu* [10]), it is more likely that litter size (i.e. increased ova available for fertilization) increases the chances of sharing paternity [39] and so may be expected to have a positive effect on male post-copulatory intrasexual competition and thus relative testes size. Larger litters/clutches also alter the ability to detect multiple paternity [4], [21], but since both multiple paternity and litter size were significant in the same model, this indicates that litter size is still having a significant positive impact on relative testes size, probably as a results of increasing litter size increasing multiple paternity rates [40]. In addition, the number of litters a female produces in each reproductive period may be important; combined with litter size, the annual total number of offspring produced by females may be an important variable in determining RTS and requires future examination to assess its importance.

### Relative Testes Size, Extra Group Paternity and Alpha Paternity

In parallel to other studies (see [41]), the rates of paternity loss to males outside groups (extra group paternity) were higher in mammals (*x*– = 22%) than in birds (13%–18%: [42]; *x*– = 13.1%: [43]), again probably reflecting higher promiscuity in mammals. Similarly to birds [17], levels of extra group paternity did not reflect social mating systems.

Relative testes size was not found to correlate to extra group paternity in mammals, contrasting with data from birds [39]. Although avian relative testes size was positively associated with extra group paternity, it varied among species with low extra group paternity rates (<30%: [44]), such that some species with high extra group paternity rates had relatively small testes. However, these results have been criticised due to methodological constraints on collection of testes size data, and a smaller more robust dataset did not find this relationship [45]. So it is unclear whether extra group paternity in birds does correlate to testes size. It is known that within-species levels of extra group paternity vary with local conditions such as density [30], [46], [47], and behaviour may be evolving faster than morphological traits [31]. Because of this, it may be that extra group paternity does not relate to testes size in birds and mammals, but this relationship requires greater examination in both birds and mammals. For mammals at least, most social groups are made up of multiple males [41], so ability of the most dominant male to dominate all reproduction (alpha paternity) may more important than paternity loss to males outside the social group.

In accordance with this prediction, there was a significant negative relationship between relative testes size and alpha paternity; relative testes size was smaller in species where dominant males gained a higher share of paternities. Alpha paternity did not vary with mating system, despite other studies showing that social structure and the number of males in a social group can affect alpha paternity levels [48], [49]. Other variables, such as female reproductive synchrony, male-female association type and ovulation mode may be confounding this result [35], [48]–[50]. Social mating system may be expected to correlate with testes size in species where alpha paternity may be important, e.g. primates [14], [27]. This also suggests that social mating system does not reflect alpha paternity across all mammals but may instead have taxa specific relationships.

## Materials and Methods

I collated data on testes mass (excluding epididymides), body mass, multiple paternity, EGP and alpha paternity from species of wild mammal populations. The latter two variables could only be collated from pair- or group-living species. For some species (*n* = 10), multiple paternity values from different populations were available (range 2–8); in these cases I used mean values in the analysis. Alpha paternity can only be calculated where the dominant male has been identified, or if only one male was present, he was assumed to be dominant. In species with single male groups, alpha paternity would therefore be related to extra group paternity. Testes mass was taken as the combined wet mass of both testes taken from healthy adult males at the peak of the reproductive season [45]. Care was taken to match testes mass and body mass from the same geographic location, but the large samples sizes advocated to calculate mean testes size were not possible in many studies [45].

For all models, I included body mass (to avoid using residuals in the model: [51]), ovulation mode and length of mating season as these were important in previous analyses of testes mass [15]. In addition, I included litter size in the analysis of testes mass and multiple paternity, to avoid biases in the detection of multiple paternity [21], though I included it as a predictor variable rather than using residuals [51]. I could not include this in the EGP and alpha paternity models as data were heavily skewed to species with single offspring. Data on all variables were collated from published sources and from the same population where possible (Appendix S1). Full models were chosen, rather than a stepwise or information theoretic (IT) approach [52]. I used a phylogenetically corrected general linear model (PGLM; for details see: [15]), using a mammalian phylogeny with branch lengths [53], [54]. In PGLM, λ is set to its maximum likelihood value rather than assuming clear-cut phylogenetic dependence/independence of data [55]. Residual outliers were identified using conditional boxplots and where these may have occurred, tested using Grubb's test [56], [57]. The phylogenetically correct residuals used in the figures were taken from the PGLM model following the stepwise insertion of prior variables in the order listed in Table 1.

I examined multiple paternity, EGP and alpha paternity rates in relation to social mating system using one-way ANOVAs. Social mating systems were classified as: *monogamous* (SM): one male, one female; *polygynous* (SP): one male, multiple females; *multi-male* (MM): multiple males, one or multiple females (after [38]). Lastly, I analysed the correlation between multiple paternity, EGP and alpha paternity rates using a Pearson's correlation. All data were transformed to meet normality assumptions. Analyses were run on the statistical package ‘R’ version 2.8.0 (R Foundation for Statistical Computing 2007) using an unpublished function written by R. Freckleton for the PGLM. I estimated effect sizes (correlation coefficient *r*, *sensu* [58]) and non-central confidence intervals (CI) from *t* values obtained from PGLMs [59].

## Supporting Information

Appendix S1 Data used for phylogenetically corrected general linear model analysis including testes mass, male body mass, multiple paternity rates, extra group paternity, and alpha paternity. Length of mating season: short (<6 months), long (≥6months); social mating systems were classified as: monogamous (SM): one male, one female; polygynous (SP): one male, multiple females; multimale (MM): multiple males, one or multiple females (after Isvaran and Clutton-Brock 2007); ovulation mode: induced (IND), spontaneous (SPO). ^1^ Indicates data taken from same study/population; ^2^ A population of feral pigs, not wild boar.
(0.42 MB DOC)

## References

[1] Birkhead TR, Møller AP (1998). Sperm competition and sexual selection..

[2] Simmons LW (2001). Sperm competition and its evolutionary consequence in the insects..

[3] Gullberg A, Tegelström H, Gelter HP (1992). DNA fingerprinting reveals multiple paternity in families of Great and Blue Tits (*Parus major* and *P*. *caeruleus*).. Hereditas.

[4] Myers EM, Zamudio KR (2004). Multiple paternity in an aggregate breeding amphibian: the effect of reproductive skew on estimates of male reproductive success.. Mol Ecol.

[5] Wolff JO, Macdonald DW (2004). Promiscuous females protect their offspring.. Trends Ecol Evol.

[6] Daly-Engel TS, Grubbs RD, Holland KN, Toonen RJ, Bowen BW (2006). Assessment of multiple paternity in single litters from three species of carcharhinid sharks in Hawaii.. Envir Biol Fishes.

[7] Uller T, Olsson M (2008). Multiple paternity in reptiles: patterns and processes.. Mol Ecol.

[8] Parker GA (1970). Sperm competition and its evolutionary consequences in the insects.. Biol Rev.

[9] Wedell N, Gage MJG, Parker GA (2002). Sperm competition, male prudence and sperm-limited females.. Trends Ecol Evol.

[10] Parker GA, Birkhead T, Møller AP (1998). Sperm competition and the evolution of ejaculates, towards a theory base.. Sperm competition and sexual selection.

[11] Møller AP (1988). Ejaculate quality, testis size and sperm competition in primates.. J Hum Evol.

[12] Parker GA, Ball MA, Stockley P, Gage MJG (1997). Sperm competition games: a prospective analysis of risk assessment.. Proc Roy Soc Lond B.

[13] Parker GA, Ball MA (2005). Sperm competition, mating rate and the evolution of testis and ejaculate sizes: a population model.. Biol Let.

[14] Kappeler PM (1997). Intrasexual selection and testis size in strepsirhine primates.. Behav Ecol.

[15] Iossa G, Soulsbury CD, Baker PJ, Harris S (2008). Sperm competition and the evolution of testes size in terrestrial mammalian carnivores.. Funct Ecol.

[16] Birkhead TR, Møller AP, Black JM (1996). Monogamy and sperm competition in birds.. Partnership in birds: the study of monogamy.

[17] Griffith SC, Owens IPF, Thuman KA (2002). Extra pair paternity in birds: a review of interspecific variation and adaptive function.. Mol Ecol.

[18] DeWoody JA, Avise JC (2001). Genetic perspectives on the natural history of fish mating systems.. J Hered.

[19] Kenagy GJ, Trombulak SC (1986). Size and function of mammalian testes in relation to body size.. J Mammal.

[20] Birkhead TR, Glover TD, Barratt LR (1999). The role of sperm competition in reproduction.. Male fertility and infertility.

[21] Ramm SA, Parker GA, Stockley P (2005). Sperm competition and the evolution of male reproductive anatomy in rodents.. Proc Roy Soc Lond B.

[22] Bryja J, Patzenhauerová H, Albrecht T, Mošanský L, Stanko M (2008). Varying levels of female promiscuity in four *Apodemus* mice species.. Behav Ecol Sociobiol.

[23] Firman RC, Simmons LW (2008). The frequency of multiple paternity predicts variation in testes size among island populations of house mice.. J Evol Biol.

[24] East ML, Burke T, Wilhelm K, Greig C, Hofer H (2003). Sexual conflicts in spotted hyenas: male and female mating tactics and their reproductive outcome with respect to age, social status and tenure.. Proc Roy Soc Lond B.

[25] Olsson M, Madsen T, Birkhead T, , Møller AP, editors.  (1998). Sexual selection and sperm competition in reptiles.. Sperm competition and sexual selection.

[26] Lack D (1968). Ecological adaptations for breeding in birds..

[27] Heske EJ, Ostfeld RS (1990). Sexual dimorphism in size, relative size of testes, and mating systems in North American voles.. J Mammal.

[28] Harcourt AH, Harvey PH, Larson SG, Short RV (1981). Testis weight, body weight and breeding system in primates.. Nature.

[29] Say L, Pontier D (2006). What determines testis size in the domestic cat (*Felis catus* L.)?. Biological Lett.

[30] Iossa G, Soulsbury CD, Baker PJ, Edwards KJ, Harris S (2009). Behavioral changes associated with a population density decline in the facultatively social red fox.. Behav Ecol.

[31] Birkhead TR, Møller AP (1993). Female control of paternity.. Trends Ecol Evol.

[32] Hosken DJ, Ward PI (2001). Experimental evidence for testis size evolution via sperm competition.. Ecol Lett.

[33] Emlen ST, Oring LW (1977). Ecology, sexual selection, and the evolution of mating systems.. Science.

[34] Ribble DO, Millar JS (1992). Intraspecific variation in testes size among northern populations of *Peromyscus*.. Funct Ecol.

[35] Soulsbury CD (2010). Ovulation mode modifies paternity monopolization in mammals.. Biol Lett.

[36] Gomendio M, Harcourt AH, Roldán ERS, Birkhead T, Møller AP (1998). Sperm competition in mammals.. Sperm competition and sexual selection.

[37] Soulsbury CD, Iossa G (Online early) The impact of ovulation mode on sperm quantity and quality in mammals.. Evol Ecol.

[38] Pitcher DE, Dunn PO, Whittingham LA (2005). Sperm competition and the evolution of testes size in birds.. J Evol Biol.

[39] Birkhead TR, Atkin L, Møller AP (1987). Copulation behaviour in birds.. Behav.

[40] Eccard JA, Wolf JBW (2009). Effects of brood size on multiple-paternity rates: a case for ‘paternity share’ as an offspring-based estimate.. Anim Behav.

[41] Isvaran K, Clutton-Brock TH (2007). Ecological correlates of extra-group paternity in mammals.. Proc Roy Soc Lond B.

[42] Westneat DF, Stewart IRK (2003). Extra-pair paternity in birds: causes, correlates and conflict.. Annu Rev Ecol Syst.

[43] Wink M, Dyrcz A (1999). Mating systems of birds: a review of molecular studies.. Acta Ornith.

[44] Møller AP, Briskie JV (1995). Extra-pair paternity, sperm competition and the evolution of testis size in birds.. Behav Ecol Sociobiol.

[45] Calhim S, Birkhead TR (2007). Testes size in birds: quality versus quantity—assumptions, errors, and estimates.. Behav Ecol.

[46] Schülke O, Kappler PM, Zichler H (2004). Small testes size despite high extra-pair paternity in the pair-living nocturnal primate *Phaner furcifer*.. Behav Ecol Sociobiol.

[47] Westneat DF, Sherman PW (1997). Density and extra-pair fertilizations in birds: a comparative analysis.. Behav Ecol Sociobiol.

[48] Cohas A, Allainé D (2009). Social structure influences extra-pair paternity in socially monogamous mammals.. Biol Lett.

[49] Ostner J, Nunn CL, Schülke O (2008). Female reproductive synchrony predicts skewed paternity across primates.. Behav Ecol.

[50] Clutton-Brock TH, Isvaran K (2006). Paternity loss in contrasting mammalian societies.. Biol Let:.

[51] Freckleton RP (2009). The seven deadly sins of comparative analysis.. J Evol Biol.

[52] Whittingham MJ, Stephens PA, Bradbury RB, Freckleton RP (2006). Why do we still use stepwise modelling in ecology and behaviour?. J Anim Ecol.

[53] Bininda-Emonds ORP, Cardillo M, Jones KE, MacPhee RDE, Beck RMD (2007). The delayed rise of present-day mammals.. Nature.

[54] Bininda-Emonds ORP, Cardillo M, Jones KE, MacPhee RDE, Beck RMD (2008). Corrigendum.. Nature.

[55] Freckleton RP, Harvey PH, Pagel M (2002). Phylogenetic analysis and comparative data: a test and review of evidence.. Am Nat.

[56] Grubbs FE (1969). Procedures for detecting outlying observations in samples.. Technometrics.

[57] Zuur AF, Ieno EN, Elphick CS (2010). A protocol for data exploration to avoid common statistical problems.. Methods Ecol Evol.

[58] Cohen J (1988). Statistical power analysis for the behavioral sciences, 2nd edn..

[59] Nakagawa S, Cuthill IC (2007). Effect size, confidence interval and statistical significance: a practical guide for biologists.. Biol Rev.

