# Supplementary material for: Genetic Patterns of Paternity and Testes Size in Mammals
Source: PLoS One. 2010 Mar 8;5(3):e9581. doi: 10.1371/journal.pone.0009581 (PMC2833195; doi:10.1371/journal.pone.0009581)
Supplement: Appendix S1 — Data used for phylogenetically corrected general linear model analysis including testes mass, male body mass, multiple paternity rates, extra group paternity, and alpha paternity. Length of mating season: short (<6 months), long (≥6months); social mating systems were classified as: monogamous (SM): one male, one female; polygynous (SP): one male, multiple females; multimale (MM): multiple males, one or multiple females (after Isvaran and Clutton-Brock 2007); ovulation mode: induced (IND), spontaneous (SPO). 1 Indicates data taken from same study/population; 2 A population of feral pigs, not wild boar. (0.42 MB DOC) [file pone.0009581.s001.doc]

**Genetic Patterns of Paternity and Testes Size in Mammals**

Carl D. Soulsbury

**Appendix S1**

Data used for PGLM analysis including testes mass, male body mass, multiple paternity rates, EGP and alpha paternity. Length of mating season: short (<6 months), long (≥6months); social mating systems were classified as: *monogamous* (SM): one male, one female; *polygynous* (SP): one male, multiple females; *multi-male* (MM): multiple males, one or multiple females (after Isvaran & Clutton-Brock 2007); ovulation mode: IND induced, SPO spontaneous. ^1^ indicates data taken from same study/population; ^2^ A population of feral pigs, not wild boar.

**APPENDIX S1**

| **Species** | **Testes mass (g)** | **Male body mass (g)** | **Mating system** | **Length of mating season (months)** | **Litter size** | **Multiple paternity (%)** | **EGP (%)** | **Alpha**  **Paternity (%)** | **Ovulation mode** | **References** |
| --- | --- | --- | --- | --- | --- | --- | --- | --- | --- | --- |
| *Acinonyx jubatus* |  |  | MM |  | 2.96^1^ | 43.5 |  |  | IND | [1, 2] |
| *Alopex lagopus* | 4.06 | 4800 | SM | Short | 3.2^2^ | 12.5 |  |  | SPO | [2-4] |
| *Alouatta seniculus* |  |  |  |  | 1 |  | 0 | 100.0 | SPO | [5-7] |
| *Antilocapra america* |  |  | SP |  |  | 44.0 |  |  |  | [8-9] |
| *Apodemus agrarius* | 0.994 | 21.8 | MM | Long | 6.5^1^ | 80.0 |  |  | SPO | [7, 10, 11] |
| *Apodemus agrarius* | 0.994 | 21.8 | MM | Long | 6.5 | 58.8 |  |  | SPO | [7, 10, 12] |
| *Apodemus flavicollis* | 0.891 | 29.1 | MM | Long | 5 | 30.0 |  |  | SPO | [7, 10, 13] |
| *Apodemus flavicollis* | 0.891 | 29.1 | MM | Long | 5 | 60.0 |  |  | SPO | [7, 10, 12] |
| *Apodemus sylvaticus* | 0.788 | 23.1 | MM | Long | 4.1 | 50.0 |  |  | SPO | [7, 10, 11] |
| *Apodemus sylvaticus* | 0.788 | 23.1 | MM | Long | 4.1 | 68.2 |  |  | SPO | [7, 10, 12] |
| *Apodemus uralensis* | 0.326 | 19.2 | MM | Long | 5 | 43.5 |  |  | SPO | [7, 10, 12] |
| *Artibeus jamaicensis* |  |  | SP |  |  |  | 21.9 | 68.8 |  | [14, 15] |
| *Artibeus watsoni* |  |  | SP |  |  |  | 73.3 | 26.7 |  | [16, 17] |
| *Canis latrans* | 15.4 | 10600 | SM | Short | 5 | 0 | 0 |  | SPO | [2, 18] |
| *Canis simensis* |  |  | SM |  |  | 33.3 | 27.6 | 91.7 |  | [19, 20] |
| *Capreolus capreolus* | 45.0 | 20500 | SP | Short |  | 15.5 |  |  | SPO | [161, 162] |
| *Castor canadensis* |  |  | SM |  |  | 55.6 |  |  |  | [21] |
| *Cavia aperea* | 3.73 | 567 | SP | Long | 3 | 26.7 |  |  | SPO | [7, 22-24] |
| *Cebus capucinus* |  |  | MM |  |  |  | 0 | 87.5 | SPO | [6, 25] |
| *Cervus elaphus* | 217 | 121800 | SP | Short | 1 |  | 37.8 | 62.2 | SPO | [26, 27] |
| *Cheirogaleus medius* |  |  | SM |  |  |  | 43.8 | 56.2 |  | [28, 29] |
| *Clethrionomys glareolus* | 0.646 | 21.8 | MM | Long | 5.3 | 35.5 |  |  | IND | **[7, 10, 23 30, 31]** |
| *Crocidura russula* | 0.04 | 14.2 | SM | Long | 2.3^1^ | 0 | 0 | 100.0 | IND | [7, 32-34] |
| *Crocuta crocuta* | 9.85 | 45000 | MM | Long | 1.65^1^ | 21.4 |  |  | SPO | [2, 35] |
| *Crocuta crocuta* | 9.85 | 45000 | MM | Long | 1.38 | 34.7 |  | 5.3 | SPO | [2, 36] |
| *Cryptomys hottentotus* | 0.58 | 104 | SM | Short | 2.6^1^ |  | 21.9 | 79.4 | IND | [37-39] |
| *Ctenomys talarum* | 0.3^1^ | 136^1^ | SP | Long | 2.6^1^ | 0 |  |  | IND | [40-43] |
| *Cynictis penicillata* | 1.98 | 900 | SP | Long | 2.5 | 28.6 |  | 45.1 | IND | [2, 172] |
| *Cynomys gunnisoni* |  |  | SP |  |  | 77.8 |  |  |  | [23, 44] |
| *Cynomys ludovicianus* | 4.00 | 1050 | SP | Short | 2.9^1^ | 2.9 |  |  | SPO | [7, 23, 45-47] |
| *Cynomys parvidens* |  |  | SP |  |  | 71.4 |  |  |  | [23, 44] |
| *Dipodymys spectablis* |  |  | SP |  |  | 65.6 |  |  |  | [48, 49] |
| *Eptesicus fuscus* |  |  | MM |  |  | 46.2 |  |  |  | [50] |
| *Erinaceous europaeus* | 2.31 | 665 | MM | Short | 5^1^ | 40.0 |  |  | IND | [10, 164,] |
| *Eulemur fulvis* | 7.78 | 2500 | SP | Short | 1 |  | 0 | 87.5 | SPO | [51-53] |
| *Felis catus* | 3.61 | 4770 | SP | Long | 3.5 | 12.5 |  |  | IND | [2, 54] |
| *Felis catus* | 3.61 | 4770 | MM | Long | 3.5 | 76.0 |  |  | IND | [2, 54] |
| *Felis catus* | 3.61 | 4770 | MM | Long | 3.78^1^ | 77.8 |  | 43.8 | IND | [2, 55] |
| *Felis catus* | 3.61 | 4770 | SP | Long | 3.08^1^ | 0 |  |  | IND | [2, 56] |
| *Felis concolor* | 14.9 | 45000 | SP | Long | 2.5^1^ | 0 |  |  | IND | [2, 57] |
| *Gorilla berengei* | 36 | 200000 | MM | Long | 1 |  | 0 | 78.0 | SPO | [23, 51, 58] |
| *Gulo gulo* | 17 | 14500 | MM | Short | 1.76^1^ | 12.5 |  |  | IND | [2, 59] |
| *Hapalemur griseus* |  |  | SM |  |  |  | 8.5 |  |  | [60] |
| *Helogale parvula* | 0.45 | 455 | MM | Long | 2.83^1^ | 19.0 | 0 | 75.6 | IND | [61-63] |
| *Hyaena hyaena* |  |  | MM |  |  | 50.0 | 30.8 |  |  | [139] |
| *Hypogeomys antimena* |  |  | SM |  |  |  | 4.2 | 95.8 |  | [23, 64] |
| *Lasiurus borealis* |  |  | MM |  |  | 20.0 |  |  |  | [65, 66] |
| *Lepus americanus* | 11.8 | 1300 | MM | Short | 3.8^1^ | 25.0 |  |  | IND | [7, 67, 68] |
| *Lycaon pictus* |  |  | MM |  |  | 10.0 |  | 89.0 |  | [2, 69] |
| *Macaca fascicularis* | 30.8 | 4400 | MM | Short | 1 |  | 0 | 67.0 | SPO | [6, 7, 71, 72] |
| *Macaca fuscata* | 70 | 12300 | MM | Short | 1 |  | 33.0 | 33.0 | SPO | [6, 7, 73, 164] |
| *Macaca sinica* |  |  | MM |  |  |  | 16.1 | 43.0 | SPO | [74] |
| *Madoqua kirki* |  |  | SM |  |  |  | 0 |  |  | [75] |
| *Marmota marmota* |  |  | SM |  |  | 27.7 | 11.1 | 84.1 | IND | [76, 77] |
| *Mastomys natalensis* | 0.972 | 59 | MM | Short | 9.9^1^ | 67.0 |  |  | SPO | [ 78-80] |
| *Meles meles* | 14.41 | 14515 | MM | Short | 1.4^1^ | 44.8 |  |  | IND | [2, 81] |
| *Meles meles* | 14.41 | 14515 | MM | Short | 1.46 | 16.1 | 45.0 |  | IND | [2, 82] |
| *Microcebus murinus* | 1.83 | 71.66 | MM | Short | 2^1^ | 65.4 |  |  | SPO | [83-86] |
| *Microtus ochrogastor* | 0.88 | 38 | SM | Long | 4.8^1^ | 55.6 |  |  | IND | [7, 87-89] |
| *Microtus pennsylvanicus* | 1.17 | 54.6 | MM | Long | 5.96 | 33.1 |  |  | IND | [7, 23, 90-92] |
| *Microtus oeconomus* | 0.42 | 35 | MM | Short | 5.28^1^ | 38.1 |  |  | IND | [7, 10, 173] |
| *Mirounga angurostris* |  |  | SP |  |  |  | 61.1 | 38.9 | SPO | [93, 94] |
| *Mirounga leonina* |  |  | SP |  |  |  | 42.0 | 58.0 | SPO | [93, 94] |
| *Mus musculus* | 0.119 | 15 | MM | Long | 5.6^1^ | 23.1 |  |  | SPO | [7,10, 95, 96] |
| *Mus musculus* | 0.159 | 22^1^ | MM | Long | 6.0^1^ | 31.8 |  |  | SPO | [7, 10, 95, 97] |
| *Mus musculus* | 0.144^1^ | 21^1^ | MM | Long | 7.6^1^ | 10.0 |  |  | SPO | [7, 10, 95, 97] |
| *Mus musculus* | 0.126^1^ | 19.4^1^ | MM | Long | 7.5^1^ | 20.0 |  |  | SPO | [7, 10, 95, 97] |
| *Mus musculus* | 0.116^1^ | 15.9^1^ | MM | Long | 5.7^1^ | 38.1 |  |  | SPO | [7, 10, 95, 97] |
| *Mus musculus* | 0.112^1^ | 18.2^1^ | MM | Long | 5.6^1^ | 5.6 |  |  | SPO | [7, 10, 95, 97] |
| *Mus musculus* | 0.123^1^ | 16^1^ | MM | Long | 5.8^1^ | 30.8 |  |  | SPO | [7, 10, 95, 97] |
| *Mus musculus* | 0.122^1^ | 15^1^ | MM | Long | 4.8^1^ | 42.9 |  |  | SPO | [7, 10, 95, 97] |
| *Mustela erminea* | 2.64 | 324 | SP | Short | 6.3^1^ | 16.7 |  |  | IND | [2, 98] |
| *Mustela vison* | 4.95 | 1710 | MM | Short | 3.16^1^ | 33.3 |  |  | IND | [2, 99] |
| *Myodes rufocanus* | 0.161 | 39 | MM | Short | 7.3^1^ | 23.2 |  |  | IND | [7, 100-103] |
| *Neotoma cinerea* |  |  | SP |  | 1.66^1^ | 0 |  |  | SPO | [7, 104-106] |
| *Neotoma fuscipes* |  |  | SP |  |  | 33.3 |  |  | SPO | [7, 106, 107] |
| *Neotoma macrotis* |  |  | SP | Long | 1.63^1^ | 20.0 |  |  | SPO | [67 106, 108] |
| *Neotoma micropus* | 2.5 | 265 | MM | Long | 2 | 66.7 |  |  | SPO | [7, 106. 174, 175] |
| *Ochotona curzoniae* | 1.62 | 147.5 | SP | Short | 6 |  | 1.7 | 69.4 | IND | [165, 166, 171] |
| *Odocoileus virgianus* | 85.5 | 44800 | MM | Short | 2^1^ | 22.2 |  |  | SPO | [7, 10, 109, 110] |
| *Oryctolagus cuniculus* | 6.06 | 2888 | MM | Short |  |  |  | 90.0 | IND | [10, 167, 168] |
| *Ovis aries* | 318.8 | 35000 | MM | Short | 2^1^ | 73.8 |  |  | SPO | [10, 111] |
| *Pan paniscus* | 250 | 45000 | MM | Long | 1 |  | 9.1 | 19.0 | SPO | [6, 71, 112] |
| *Pan troglodytes* | 118 | 44300 | MM | Long | 1 |  | 7.1 | 38.0 | SPO | [6 , 71, 113] |
| *Panthera leo* | 55 | 188000 | SP | Long | 2.75 | 14.2 | 0 | 100.0 | IND | [2,7, 114] |
| *Papio cynocephalus* | 52 | 243200 | MM | Long | 1 |  | 0 | 81.0 | SPO | [6, 61, 115] |
| *Peromyscus californicus* | 9.6 | 52.3 | SM | Long | 2.03 | 0 | 0 | 100.0 | SPO | [7, 116-118] |
| *Peromyscus crinitus* |  |  | MM |  |  | 20.0 |  |  | SPO | [7, 119, 120] |
| *Peromyscus leucopus* | 0.46 | 22.2 | MM | Long | 3.74^1^ | 11.5 |  |  | SPO | [7, 121-123] |
| *Peromyscus maniculatus* | 0.393 | 19.5 | MM | Long | 4.78^1^ | 10.3 |  |  | SPO | [7, 10, 23, 124, 125] |
| *Phaner furcifer* |  |  | SM |  |  |  | 57.1 | 42.9 |  | [126, 127] |
| *Potos flavus* |  |  | MM |  |  |  |  | 100.0 |  | [169] |
| *Procyon lotor* | 13.72 | 5130 | MM | Long | 3.2^1^ | 87.5 |  |  | IND | [2, 128] |
| *Propithecus verreauxi* |  |  | MM |  |  |  | 0 | 100.0 | SPO | [129, 130] |
| *Rangifer tarandus* | 47.6 | 110500 | MM | Short |  |  |  | 50.7 | SPO | [63, 178, 179] |
| *Saccopteryx bilineata* |  |  | SP |  |  |  | 69.9 |  |  | [131] |
| *Sanguinus mystax* |  |  | MM |  |  |  | 0 | 92.0 |  | [6, 132] |
| *Semnopithecus entellus* | 11.5 | 19200 | MM | Short | 1 |  | 14.3 | 72.2 | SPO | [71, 133, 134] |
| *Sorex araneus* | 0.17 | 11.9 | MM | Long | 6.5 | 88.9 |  |  | IND | [34, 135, 136] |
| *Spermophilus beecheyi* | 9.05 | 840 | SP | Short | 7.1^1^ | 88.9 |  |  | IND | [7, 10, 23, 137] |
| *Spermophilus beldingi* | 3.5 | 370 | SP | Short | 4.60 | 77.8 |  |  | IND | [7, 10, 23, 138] |
| *Spermophilus parry* |  |  |  |  |  | 10.0 |  |  | IND | [163] |
| *Suricata suricatta* | 1.3 | 731 | SM | Long | 3^1^ | 31.3 | 9.3 | 77.0 | IND | [2, 140] |
| *Sus scrofa* |  |  | SP | Short | 5.56^1^ | 11.1 |  |  | SPO | **[141-143, 177]** |
| *Sus scrofa^2^* |  |  | SP | Long | 6.58 | 77.0 |  |  | SPO | [144, 145] |
| *Sus scrofa* |  | 75000 | SP | Short | 4.05^1^ | 9.5 |  |  | SPO | [176, 177] |
| *Tamias amoenus* | 1.22^1^ | 51^1^ | MM | Short | 5.32 | 91.7 |  |  | SPO | [7, 23, 146, 147] |
| *Tamiasciurus hudsonicus* | 2.92 | 186 | MM | Short | 3^1^ | 71.0 |  |  | SPO | [23, 148, 149] |
| *Tamiasciurus hudsonicus* | 2.92 | 186 | MM |  | 3 | 83.3 |  |  | SPO | [23, 148, 150] |
| *Tupaia tana* |  |  | SM | Long | 2^1^ | 32.0 | 50.0 | 52.0 | IND | [151-152] |
| *Urocyon cinereoargenteus* | 5.17 | 3700 | SM | Short | 3.3 | 14.3 |  |  | SPO | [2, 153] |
| *Urocyon littoralis* |  |  | SM |  |  |  |  | 75.0 |  | [170] |
| *Ursus americanus* | 38.9 | 102287 | MM | Short | 2.4 | 50.0 |  |  | IND | [2, 7, 154] |
| *Ursus americanus* | 38.9 | 102287 | MM | Short | 2.4 | 20.0 |  |  | IND | [2, 7, 155] |
| *Ursus arctos* | 153.4 | 312000 | MM | Short | 2 | 13.3 |  |  | IND | [2, 7, 156] |
| *Ursus arctos* | 153.4 | 312000 | MM | Short | 2 | 28.1 |  |  | IND | [2, 7, 157] |
| *Vulpes velox* |  |  | SM | Short | 3.1 |  | 52.6 | 47.4 | SPO | [7, 158] |
| *Vulpes vulpes* | 12.36 | 6260 | SP | Short | 3.5^1^ | 37.5 | 77.0 | 13.3 | SPO | [2, 159] |
| *Vulpes vulpes* | 12.36 | 6260 | SP | Short | 2.9^1^ | 0 | 37.5 |  | SPO | [2, 160] |

**References**

1. Gotteli D, Wang J, Bashir S, Durant SM (2007) Genetic analysis reveals promiscuity among female cheetahs. Proc Roy Soc Lond B 274: 1993-2001.
2. Iossa G, Soulsbury CD, Baker PJ, Harris S (2008) Sperm competition and the evolution of testes size in terrestrial mammalian carnivores. Funct Ecol 22: 655-662.
3. **Gauthie****r G, Bêty** **J, Giroux J-F, Rochefort L (2004)** Trophic interactions in a high **arctic** snow goose colony. Int Comp Biol 44: 119-129.
4. Carmichael LE, Szor G, Berteaux D, Giroux MA, Cameron C, Strobeck C (2007) Free love in the far north: plural breeding and polyandry of arctic foxes (*Alopex lagopus*) on Bylot Island, Nunavut. Can J Zool 85: 338-343.
5. Pope T (1990) The reproductive consequences of male cooperation in the red howler monkey: paternity exclusion in multi-male and single-male troops using genetic markers. Behav Ecol Sociobiol 27: 439–446.
6. Nunn CL (1999) The number of males in primate social groups: a comparative test of the socioecological model. Behav Ecol Sociobiol 46: 1-13.
7. Hayssen V, van Tienhoven A, van Tienhoven A (1993) Asdell's patterns of mammalian reproduction. Comstock, London.
8. Byers JA, Moodie JD (1990) Sex-specific maternal investment in pronghorn, and the question of a limit on differential provisioning in ungulates. Behav Ecol Sociobiol 26: 157-164.
9. Carling MD, Wiseman PA, Byers JA (2003) Microsatellite analysis reveals multiple paternity in a population of wild pronghorn antelopes (Antilocapra americana). J Mammal 84: 1237-1243.
10. Kenagy GJ, Trombulak SC (1986) Size and function of mammalian testes in relation to body size. J Mammal 67: 1-22.
11. Baker RJ, Makova KD, Chesser RK (1999) Microsatellites indicate a high frequency of multiple paternity in *Apodemus* (Rodentia). Mol Ecol 8: 107-111.
12. Bryja J, Patzenhauerová H, Albrecht T, Mošanský L, Stanko M, Stopka P (2008) Varying levels of female promiscuity in four *Apodemus* mice species. Behav Ecol Sociobiol 63: 251-260.
13. Gryczyńska-Siemiątkowska A, Gortat T, Kozakiewicz A, Rutkowski R, Pomorski J, Kozakiewicz M (2008) Multiple paternity in a wild population of the yellow-necked mouse *Apodemus flavicollis*. Acta Therio 53: 251-258.
14. Ortega J, Maldonado JE, Wilkinson GS, Arita HT, Fleischer RC (2003) Male dominance, paternity, and relatedness in the Jamaican fruit-eating bat (*Artibeus jamaicensis*). Mol Ecol 12: 2409-2415.
15. McCracken GF, Wilkinson GS (2000) Bat mating systems. In: Crichton EG, Krutzsch PH, editors. Reproductive biology of bats. Sand Diego: Academic Press. pp. 321-362.
16. Chaverri G, Gambas-Rios M, Kunz TH (2007) Range overlap and association patterns in the tent-making bat *Artibeus watsoni.* Anim Behav 73: 157-164.
17. Chaverri G, Schneider CJ, Kunz TH (2008) Mating system of the tent-making bat Artibeus watsoni (Chiroptera: Phyllostomidae). J Mammal 89: 1361-1371.
18. Hennessy C (2007) Genetic aspects of a coyote population in a suburban landscape. MSc thesis, Ohio State University, Ohio.
19. Sillero-Zubiri C, Gottelli D, Macdonald DW (1996) Male philopatry, extra-pack copulations and inbreeding avoidance in Ethiopian wolves (*Canis simensis*). Behav Ecol Sociobiol 38: 331-340.
20. Randall DA, Pollinger JP, Wayner RK, Tallents LA, Johnson PJ, Macdonald DW (2007) Inbreeding is reduced by female-biased dispersal and mating behavior in Ethiopian wolves. Behav Ecol 18: 579-589.
21. Crawford JC, Liu Z, Nelson TA, Nelson CK, Bloomquist CK (2008) Microsatellite analysis of mating and kinship in beavers (Castor canadensis). J Mammal 89: 575-581.
22. Cooper TG, Weydert S, Yeung CH, Kunzl C, Sachser N (2000) Maturation of epididymal spermatozoa in the nondomesticated guinea pigs *Cavia aperea* and *Galea musteloides.* J Androl 21: 154-163.
23. Waterman JM (2007) Male mating strategies in rodents. In: Wolff JO, Sherman PW, editors. Rodent societies: an ecological and evolutionary perspective. Chicago: Academic Press. pp. 27-41.
24. Asher M, Lippmann T, Epplen JT, Kraus C, Trillmich F, Sachser N (2008) Large males dominate: ecology, social organization, and mating system of wild cavies, the ancestors of the guinea pig. Behav Ecol Sociobiol 62: 1509-1521.
25. Jack K, Fedigan L (2006) Why be alpha male? Dominance and reproductive success in wild white-faced capuchins (*Cebus capuchinus*). In New perspectives in the study of Mesoamerican primates: Distribution, ecology, behavior and conservation, (A. Estrada, P. Garber, L. Luecke, M. Pavelka eds), pp. 367-363. Springer: New York.
26. Guinness F, Lincoln GA, Short RV (1971) The reproductive cycle of the female red deer, *Cervus elaphus* L. J Reprod Fert 2: 427-438.
27. Clutton-Brock TH, Isvaran K (2006) Paternity loss in contrasting mammalian societies. Biol Lett 2: 513-516.
28. Fietz J (1999) Monogamy as a rule rather than exception in nocturnal lemurs: the case of the fat-tailed dwarf lemur, *Cheirogaleus medius*. Ethology 105: 255-272.
29. Fietz J, Zischler H, Schwiegk C, Tomiuk J, Dausmann KH, Ganzhorn JU (2000) High rates of extra-pair young in the pair-living fat-tailed dwarf lemur, *Cheirogaleus medius.* Behav Ecol Sociobiol 49: 81-17.
30. **Ödberg FO (1984)** Some data on the fertility of bank voles (*Clethrionomys glareolus britannicus*) in the laboratory supporting the hypothesis of induced ovulation. Lab Anim **18**: 33-35.
31. Ratkiewicz M, Borkowska A (2000) Multiple paternity in the bank vole (*Clethrionomys glareolus*): field and experimental data. Z Säugetier 65: 6-14.
32. Bedford JM, Phillips DM, Moyer-Lev H (1997) Novel sperm crypts and behavior of gametes in the fallopian tube of the white-toothed shrew, *Crocidura russula* Monacha. J Exp Zool 277: 262-273.
33. Bouteiller C, Perrin N (2000) Individual reproductive success and effective population size in the greater white-toothed shrew *Crocidura russula*. Proc Roy Soc Lond B 267: 701-705.
34. **Parapanov** R, N**usslé S, Vogel P (2007)** Cycle length of spermatogenesis in shrews (Mammalia: Soricidae) with high and low metabolic rates and different mating systems. Biol Reprod 76: 833-840.
35. Engh AL, Funk SM, Van Horn RC, Scribner KT, Bruford MW et al. 2002 Reproductive skew among males in a female-dominated mammalian society. Behav Ecol 13: 193- 200.
36. East ML, Burke T, Wilhelm K, Greig C, Hofer H (2003) Sexual conflicts in spotted hyenas: male and female mating tactics and their reproductive outcome with respect to age, social status and tenure. Proc Roy Soc Lond B 270: 1247-1254.
37. van Rensburg LJ, Bennett NC, van der Merwe M, Schoeman AS (2002) Seasonal reproduction in the highveld mole-rat *Cryptomys hottentotus pretoriae* (Rodentia: Bathyergidae). Can J Zool 80: 810-820.
38. Bishop JM, Jarvis JUM, Spinks RC, Bennett NC, O’Ryan C (2004) Molecular insight into patterns of colony composition and paternity in the common mole-rat *Cryptomys hottentotus hottentotus*. Mol Ecol 13: 1217-1229.
39. Jackson CR, Bennett NJ (2005) Is the natal mole-rat (Cryptomys hottentotus natalensis) a spontaneous or induced ovulator? J Mammal 68: 1-6.
40. Weir BJ (1974) Reproductive characteristics of hystricomorph rodents. Symp Zool Soc Lond 34: 265-301.
41. Malizia AI, Busch C (1991) Reproductive parameters and growth in the fossorial rodent *Ctenomys talarum* (Rodentia, Octodontidae). Mammalia 55: 293-305.
42. Zenuto RR, Lacey EA, Busch C (1999) DNA fingerprinting reveals polygyny in the subterranean rodent *Ctenomys talarum*. Mol. Ecol. 8, 1529-1532.
43. Zenuto RR, Malizia AI, Busch C (1999) Sexual size dimorphism, testes size and mating system in two populations of *Ctenomys talarum* (Rodentia: Octodontidae). J Nat Hist 33: 305-314.
44. Haynie ML, Van Den Bussche RA, Hoogland JL, Gilbert DA (2003) Parentage, multiple paternity, and breeding success in Gunnison's and Utah prairie dogs. J Mammal 84: 1244-1253.
45. Anthony A (1953) Seasonal reproductive cycle in the normal and experimentally treated male prairie dog, *Cynomys ludovicianus*. J Morphol 93: 331-370.
46. Foreman D (1962) The normal reproductive cycle of the female prairie dog and the effects of light. Anat Rec 142: 391-405.
47. Hoogland JL, Foltz DW (1982) Variance in male and female reproductive success in a harem-polygynous mammal, the black-tailed prairie dog (Sciuridae: *Cynomys ludovicianus*). Behav Ecol Sociobiol 11: 155-163.
48. Randall JA (1991) Mating strategies of a nocturnal, desert rodent (*Dipodomys spectabilis*). Behav Ecol Sociobiol 28: 215-220.
49. Waser PM, De Woody JA (2006) Multiple **paternity** in a philopatric rodent: the interaction of competition and choice. Behav Ecol 17: 971-978.
50. Vonhof MJ, Barber D, Fenton MB, Strobeck C (2006) A tale of two siblings: multiple paternity in big brown bats (*Eptesicus fuscus*) demonstrated using microsatellite markers. Mol Ecol 15: 241-247.
51. Harcourt AH, Harvey PH, Larson SG, Short RV (1981) Testis weight, body weight and breeding system in primates. Nature **293**: 55-57.
52. Ostner J, Kappeler PM (1999) Central males instead of multiple pairs in redfronted lemurs, *Eulemur fulvus rufus* (Primates, Lemuridae)? Anim Behav 58: 1069-1078.
53. Wimmer B, Kappeler PM (2002) The effects of sexual selection and life history on the genetic structure of redfronted lemur, *Eulemur fulvus rufus*, groups. Anim Behav 64 557–568.
54. Say L, Pontier D, Natoli E (1999) High variation in multiple paternity of domestic cats (*Felis catus* L.) in relation to environmental conditions. Proc Roy Soc Lond B 266: 2071-2074.
55. Natol E, Schmid M, Say L, Pontier D (2007) Male reproductive success in a social group of urban feral cats (*Felis catus* L.). Ethology 113: 283-289.
56. Say L, Devillard S, Natoli E, Pontier D (2002) The mating system of feral cats (*Felis catus* L.) in a sub-Antarctic environment. Polar Biol 25: 838-842.
57. Murphy KM (1998) The ecology of the cougar (*Puma concolor*) in the Northern Yellowstone ecosystem: interactions with prey, bears, and humans, PhD thesis, University of Idaho, Moscow, Idaho.
58. Bradley BJ, Robbins MM, Williamson EA, Steklis HD, Steklis NG et al. (2005) Mountain gorilla tug-of-war: silverbacks have limited control over reproduction in multimale groups. Proc Natl Acad Sci USA 102: 9418–9423.
59. Hedmark E, Persson J, Segerström P, Landa A, Ellegren H (2007) Paternity and mating system in wolverines *Gulo gulo*. Wildlife Biol. 13 Supplement 2: 13-30.
60. Nievergelt CM, Mutschler T, Feistner AT, Woodruff DS (2002) Social system of the Alaotran gentle lemur (*Hapalemur griseus alaotrensis*): genetic characterization of group composition and mating system. Am J Primatol 57: 157-176.
61. Keane B, Waser PM, Creel SR, Creel NM, Elliott LF, Minchella DJ (1994) Subordinate reproduction in dwarf mongooses. Anim Behav 47: 65-75.
62. Keane B, Creel SR, Waser PM (1996) No evidence of inbreeding avoidance or inbreeding depression in a social carnivore. Behav Ecol 7: 480-489.
63. Anderson MJ, Nyholt J, Dixson AF (2004) Sperm competition affects the structure of the mammalian vas deferens. J Zool 264: 97-103.
64. Sommer S, Tichy H (2002) Major histocompatibility complex (MHC) class II polymorphism and paternity in the monogamous *Hypogeomys antimena*, the endangered, largest endemic Malagasy rodent. Mol Ecol 8: 1259-1272.
65. Spradling KD, Stangl FB, Cook WB (2003) Evidence for a case of multiple paternity in the red bat (*Lasiurus borealis*) as indicated by DNA fingerprinting. Occ Pap Mus Tex Uni 224: 1-10
66. Cryan PM (2008) Mating behavior as a possible cause of bat fatalities at wind turbines. J Wild Manage 72: 845-849
67. Newson J (1964) Reproduction and prenatal mortality of snowshoe hares on Manitoulin Island, Ontario. Can J Zool 42: 987-1005.
68. Burton C (2002) Microsatellite analysis of multiple paternity and male reproductive success in the promiscuous snowshoe hare. Can J Zool 80: 1948-1956.
69. Girman DJ, Mills MGL, Geffen E, Wayne RK (1997) A molecular genetic analysis of social structure, dispersal, and interpack relationships of the African wild dog (*Lycaon pictus*). Behav Ecol Sociobiol 40: 187-198.
70. DELETED
71. Møller AP (1988) Ejaculate quality, testis size and sperm competition in primates. J Hum Evol 17: 479-488.
72. Engelhardt A, Heistermann M, Hodges J, Nürnberg P, Niemitz C (2006) Determinants of male reproductive success in wild long-tailed macaques (*Macaca fascicularis*)—male monopolisation, female mate choice or post-copulatory mechanisms? Behav Ecol Sociobiol 59: 740–752.
73. Soltis J, Thomsen R, Takenaka O (2001) The interaction of male and female reproductive strategies and paternity in wild Japanese macaques, *Macaca fuscata*. Anim Behav 62: 485-494.
74. Keane B, Dittus WPJ, Melnic DJ (1997) Paternity assessment in wild groups of toque macaques *Macaca sinica* at Polonnaruwa, Sri Lanka using molecular markers. Mol Ecol 6: 267-282.
75. Brotherton PNM, Pemberton JM, Komers PE, Malarky G (1997) Genetic and behavioural evidence of monogamy in a mammal, Kirk's dik-dik (*Madoqua kirkii*). Proc Roy Soc Lond B 264: 675-681.
76. Saboureau M, Lacroix A (1994) Seasonal endocrine profiles in the alpine marmot (*Marmota marmota*). From: Le Berre, M. & Ramousse, R., Report on the second international conference on marmots. Ibex 2: 23-48
77. Cohas A, Yoccoz NG, Da Silva A, Goossens B, Allaine D (2006) Extra-pair paternity in the monogamous alpine marmot (*Marmota marmota*): the roles of social setting and female mate choice. Behav Ecol Sociobiol 59: 597-605
78. Breed WG, Taylor J (2000) Body mass, testes mass, and sperm size in murine rodents. J Mammal 81: 758-768.
79. Skinner JD, Chimimba CT (2005) The mammals of the southern African subregion. 3rd edn. Cambridge: Cambridge University Press.
80. Kennis J, Sluydts V, Leirs H, van Hooft WF (2008) Polyandry and polygyny in as African rodent species, *Mastomys natalensis*. Mammalia 72: 150-160.
81. Dugdale HL, Macdonald DW, Pope LC, Burke T (2007) Polygynandry, extra-group paternity and multiple-paternity litters in European badger (*Meles meles*) social groups. Mol Ecol 16: 5294-5306.
82. Carpenter PJ, Pope LC, Greig C, Dawson DA, Rogers LM, et al. (2005) Mating system of the Eurasian badger, *Meles meles*, in a high density population. Mol Ecol 14: 273-284.
83. Fietz J (1999) Mating system of *Microcebus murinus*. Am J Primat 48: 127-133.
84. Aslam H, Schneiders A, Perret M, Weinbauer GF, Hodges JK (2002) Quantitative assessment of testicular germ cell production and kinematic and morphometric parameters of ejaculated spermatozoa in the grey mouse lemur, *Microcebus murinus*. Reprod 123: 323-332.
85. Eberle M, Kappeler PM (2002) Mouse lemurs in space and time: A test of the socioecological model. Behav Ecol Sociobiol 51: 131–139.
86. Eberle M, Kappeler PM (2004) Selected polyandry: female choice and inter-sexual conflict in a small nocturnal solitary primate (*Microcebus murinus*)*.* Behav Ecol Sociobiol 57: 91-100.
87. Richmond M, Conaway C (1969) Induced ovulation and oestrus in *Microtus ochrogaster*. J Reprod Fertil Suppl 6: 357–376.
88. Rose RK, Gaines MS (1978) The reproductive cycle of *Microtus ochrogaster* in Eastern Kansas. Ecol Mono 48: 21-42.
89. Solomon NG, Keane B, Knoch LR, Hogan PJ (2004) Multiple paternity in socially monogamous prairie voles (*Microtus ochrogaster*) Can. J Zool 82: 1667-1671.
90. Clullow FV, Mallory FF (1970) Oestrus and induced ovulation in the meadow vole, *Microtus pennsylvanicus*. J Reprod Fertil 23: 341-343.
91. Pierce JD, Ferguson B, Salo AL, Sawrey DK, Shapiro LE, et al. (1990) Patterns of sperm allocation across successive ejaculates in four species of voles (Microtus). J Reprod Fertil 88: 141-149.
92. Boonstra R, Xia X, Pavone L (1993) Mating system of the meadow vole, *Microtus pennsylvanicus*. Behav Ecol 4: 83-89.
93. Hoelzel AR, Le Boeuf BJ, Reiter J, Campagna C (1999) Alpha-male paternity in elephant seals. Behav Ecol Sociobiol 46: 298-306.
94. Larivière S, Ferguson SH (2002) On the evolution of the mammalian baculum: vaginal friction, prolonged intromission or induced ovulation? Mamm Rev 32: 283–294.
95. Allen E (1922) The oestrous cycle in the mouse. Am J Anat 30: 297-371.
96. Dean MD, Ardlie KG, Nachman MW (2006) The frequency of multiple paternity suggests that sperm competition is common in house mice (*Mus domesticus*). Mol Ecol 15: 4141-4151.
97. Firman RC, Simmons LW (2008) The frequency of multiple paternity predicts variation in testes size among island populations of house mice. J Evol Biol 21: 1524-1533.
98. Holland OJ, Gleeson DM (2005) Genetic characterization of blastocysts and the identification of an instance of multiple paternity in the stoat (*Mustela erminea*). Cons Genet 6: 855-858.
99. Yamaguchi N, Sarno RJ, Johnson WE, O'Brien SJ, Macdonald DW (2004) Multiple paternity and reproductive tactics of free-ranging American minks, *Mustela vison*. J Mammal 85: 432-439.
100. Westlin, L.M. 1982 Sterile matings at the beginning of the breeding season in *Clethrionomys rufocanus* and *Microtus agrestis*.
     Can. J. Zool. 60, 2568-2571.
101. Kawata M (1985) Mating system and reproductive success in a spring population of the red-backed vole, *Clethrionomys rufocanus* bedfordiae. Oikos 45: 181-190.
102. Heikkilä J, Kaarsalo K, Mustonen O, Pekkarinen P (1993) Influence of predation risk on early development and maturation in three species of *Clethrionomys* voles. Annal Zool Fenn 30: 153–161.
103. Ishibashi Y, Saitoh T (2008) Effect of local density of males on the occurrence of multimale mating in gray-sided voles (Myodes rufocanus). J Mammal 89: 388-397.
104. Topping MG, Millar JS (1996) Spatial distribution in the bushy-tailed wood rat (*Neotoma cinerea*) and its implications for the mating system. Can J Zool 74: 565-569.
105. Topping MG, Millar JS (1999) Mating patterns and reproductive success in the bushy-tailed woodrat (*Neotoma cinerea* ), as revealed by DNA fingerprinting. Behav Ecol Sociobiol 43: 115-124.
106. Poor A (2005) "Neotominae" (On-line), Animal Diversity Web. Accessed January 30, 2009 at <http://animaldiversity.ummz.umich.edu/site/accounts/information/Neotominae.html>.
107. McEachern MB, McElreath RL, Van Vuren DH, Eadie JM (2008) Another genetically promiscuous ‘polygynous’ mammal: mating system variation in *Neotoma fuscipes*. Anim Behav 77: 449-455.
108. Matocq MD (2004) Reproductive success and effective population size in woodrats (*Neotoma macrotis*). Mol Ecol 13: 1635-1642.
109. White LM, Hosack DA, Warren RJ, Fayrer-Hosken RA (1995) Influence of mating on duration of estrus in captive white-tailed deer. J Mammal 76: 1159-1163.
110. Sorin AB (2004) Paternity assignment for white-tailed deer (*O*docoileus virginianus): mating across age classes and multiple paternity. J Mammal 85: 356-362.
111. Pemberton JM, Coltman DW, Smith, JA, Pilkington JG (1999) Molecular analysis of a promiscuous, fluctuating mating system. Biol J Lin Soc 68: 289-301.
112. Gerloff U, Hartung B, Fruth B, Hohmann G, Tautz D (1999) Intracommunity relationships, dispersal patterns and paternity success in a wild living community of bonobos (*Pan paniscus*) determined from DNA analysis of faecal samples. Proc Roy Soc Lond B 266: 1189–1195.
113. Vigilant L, Hofreiter M, Siedel H, Boesch C (2001) Paternity and relatedness in wild chimpanzee communities. Proc Natl Acad Sci USA 98: 12890–12895.
114. Gilbert DA, Packer C, Pusey AE, Stephens JC, O'Brien SJ (1991) Analytical DNA fingerprinting in lions: parentage, genetic diversity, and kinship. J Hered 82: 378- 386.
115. Alberts S, Watts H, Altmann J (2003) Queuing and queue-jumping: long-term patterns of reproductive skew in male savannah baboons, *Papio cynocephalus*. Anim Behav 65: 821–840.
116. Kenney AM, Lanier DL, Dewsbury DA (1977) Effects of vaginal-cervical stimulation in seven species of muroid rodents. J Reprod Fert 49: 305-309.
117. Ribble DO (1991) The monogamous mating system of *Peromyscus californicus* as revealed by DNA fingerprinting. Behav Ecol Sociobiol 29: 161-166.
118. Nelson RJ, Gubernick DJ, Blom JM (1995) Influence of photoperiod, green food, and water availability on reproduction in male California mice (*Peromyscus californicus*). Phys Behav 57: 1175-1180.
119. Johnson DW, Armstrong DM (1987) *Peromyscus crinitus.* Mamm Spec 287: 1-8.
120. Shurtliff QR, Pearse DE, Rogers DS (2005) Parentage analysis of the canyon mouse (*P*eromyscus crinitus): evidence for multiple paternity. J Mammal 86: 531-540.
121. Lackey JA, Huckaby DG, Ormiston BG (1985) *Peromyscus leucopus*. Mamm Spec 247: 1-10.
122. Xia X, Millar JS (1991) Genetic evidence of promiscuity in *Peromyscus leucopus.* Behav Ecol Sociobiol 28: 171-178.
123. Ribble DO, Millar JS (1992) Intraspecific variation in testes size among northern populations of *Peromyscus*. Funct Ecol 6: 455-459.
124. Bradley EL, Terman CR (1979) Ovulation in *Peromyscus maniculatus bairdi* under laboratory conditions. J Mammal 60: 543–549.
125. Ribble DO, Millar JS (1996) The mating system of northern populations of *Peromyscus maniculatus* as revealed by radiotelemetry and DNA fingerprinting. Ecoscience 3: 423-428
126. Schülke O, Kappeler PM (2003) So near and yet so far: territorial pairs but low cohesion between pair partners in a nocturnal lemur, *Phaner furcifer.* Anim Behav 65: 331-343.
127. Schülke O, Kappeler PM, Zischler H (2004) Small testes size despite high extra-pair paternity in the pair-living nocturnal primate *Phaner furcifer*. Behav Ecol Sociobiol 55: 293-301.
128. Nielsen CLR, Nielsen CK (2007) Multiple paternity and relatedness in southern Illinois raccoons (*Procyon lotor*). J Mammal 88:441-447.
129. Brockman DK (1999) Reproductive behavior of female *Propithecus verreauxi* at Beza Mahafaly, Madagascar. Int J Primat 20: 375-398.
130. Kappeler P, Schäffler L (2006) Reproductive skew among male verreaux sifakas (*Propithecus verreauxi*) and the evolution of female dominance in Malagasy primates. Proceedings of the 11th Congress of the International Society for Behavioral Ecology; 2006 July 23–29; Tours (France). Paris: Centre National de la Recherche Scientifique.
131. Heckel G, Von Helversen O (2003) Genetic mating system and the significance of harem associations in the bat *Saccopteryx bilineata*. Mol. Ecol. 12, 219-227.
132. Huck M, Löttker P, Böhle U-R, Heymann E (2005) Paternity and kinship patterns in polyandrous moustached tamarins (*Sanguinus mystax*). Am J Phys Anthropol 127: 449–464.
133. Heistermann M, Ziegler T, van Schaik CP, Launhardt K, Winkler P, Hodges JK (2001) Loss of oestrus, concealed ovulation and paternity confusion in free-ranging Hanuman langurs. Proc Roy Soc Lond B. 268: 2445-2451.
134. Launhardt K, Borries C, Hardt C, Epplen C, Winkler P (2001) Paternity analysis of alternative male reproductive routes among the langurs (*Semnopithecus entellus*) of Ramnagar. Anim Behav 61: 53–64.
135. Brambell FWR (1935) Reproduction in the common shrew (*Sorex araneus* Linnaeus). I. The oestrous cycle of the female. Phil Trans Roy Soc B 225: 1-49.
136. Stockley P, Searle JB, Macdonald DW, Jones CS (1993) Female multiple mating behaviour in the common shrew as a strategy to reduce inbreeding. Proc Roy Soc Lond B 254: 173-179.
137. Boellstorff DE, Owings DH, Penedo MCT, Hersek MJ **(**1994) Reproductive behaviour and multiple paternity of California ground squirrels. Anim Behav 47: 1057-1064.
138. Hanken J, Sherman PW (1981) Multiple paternity in Belding's ground squirrel litters. Science 212: 351-353.
139. Wagner AP, Creel S, Frank LG, Kalinowski ST (2007) Patterns of relatedness and parentage in an asocial, polyandrous striped hyena population. Mol Ecol 16: 4356-4369.
140. Griffin AS, Pemberton JM, Brotherton PNM, McIlrath G, Gaynor D, et al. (2003) A genetic analysis of breeding success in the cooperative meerkat (*Suricata suricatta*). Behav Ecol 14: 472-480.
141. **FernáNdez-Llario P, Carranza** **J, Mateos-Quesada P (1999)** Sex allocation in a polygynous mammal with large litters: the wild boar. Anim Behav 58: 1079-1084.
142. Rosell C, Fern**áN**dez-Llario P, Herrero J (2001) El jabalí (*Sus scrofa* Linnaeus, 1758). Galemys **13:** 1–25.
143. **Delgado R, FernáNdez-Llario P, Azevedo M, Beja-Pereira A, Santos P (2008)** Paternity assessment in free-ranging wild boar (*Sus scrofa*) – Are littermates full-sibs? Mamm Biol 73: 169-176.
144. Saunders G (1993) The demography of feral pigs (*Sus scrofa*) in Kosciusko National Park, New South Wales. Wild Res 20: 559–569.
145. Spencer PBS, Lapidge SJ, Hampton JO, Pluske JR (2005) The sociogenetic structure of a controlled feral pig population. Wild Res 32: 297–304.
146. Schulte-Hostedde AI, Millar JS (2004) Intraspecific variation of testis size and sperm length in the yellow-pine chipmunk ( *Tamias amoenus*): implications for sperm competition and reproductive success. Behav Ecol Sociobiol 55: 272-277.
147. Schulte-Hostedde AI, Millar JS, Gibbs HL (2004) Sexual selection and mating patterns in a mammal with female-biased sexual size dimorphism. Behav Ecol 15: 351-356.
148. Layne JL (1954) The biology of the red squirrel *Tamiasciurus hudsonicus loquax* in central New York. Ecol Mono 24: 227-267.
149. Lane JE, Boutin S, Gunn MR, Slate J, Coltman DW (2008) Female multiple mating and paternity in free-ranging North American red squirrels. Anim Behav 75: 1927-1937.
150. Bonnano VL, Schulte-Hostedde AI (2009) Sperm competition and ejaculate investment in red squirrels (*Tamiasciurus hudsonicus*). Behav Ecol Sociobiol 63: 835-846.
151. Conaway CH, Sorenson MW (1966) Reproduction in tree shrews. Symp Zool Soc London 15: 471-492.
152. Munshi-South J (2007) Extra-pair paternity and the evolution of testis size in a behaviorally monogamous tropical mammal, the large treeshrew (*Tupaia tana*). Behav Ecol Sociobiol 62: 201-212.
153. Glenn JLW, Civitello DJ, Lance SL (2009) Multiple paternity and kinship in the gray fox (*Urocyon cinereoargenteus*). Mamm. Biol. (DOI 10.1016/j.mambio.2008.10.003)
154. Schenk A, Kovacs KM (1995) Multiple mating between black bears revealed by DNA fingerprinting. Anim Behav 50: 1583-1490.
155. Onorato DP, Hellgren EC, Van Den Bussche RA, Skiles JR (2004) Paternity and relatedness of American black bears recolonizing a desert montane island. Can J Zool 82: 1201-1210.
156. Bellemain E, Swenson JE, Taberlet P (2006) Mating strategies in relation to sexually selected infanticide in a non-social carnivore: the brown bear. Ethology 112: 238-246.
157. Craighead L, Paetkau D, Reynolds HV, Vyse ER, Strobeck C (1995) Microsatellite analysis of paternity and reproduction in Arctic grizzly bears. J Hered 86: 255-261.
158. Kitchen AM, Gese EM, Waits LP, Karki SM, Schauster ER (2006) Multiple breeding strategies in the swift fox, *Vulpes velox.* Anim Behav 71, 1029-1038.
159. Baker PJ, Funk SM, Bruford MW, Harris S (2004) Polygynandry in a red fox population: implications for the evolution of group living in canids? Behav Ecol 15:766–778.
160. Iossa G, Soulsbury CD, Baker PJ, Edwards KJ, Harris S (2009) Behavioral changes associated with a population density decline in the facultatively social **red fox. Behav Ecol 20: 385-395.**
161. Sempéré AJ, Mauget R, Chemineau P (1992) Experimental induction of luteal cyclicity in roe deer (*Capreolus capreolus*). J Reprod Fert 96: 379-384.
162. Vanpé C, Kjellander P, Gaillard JM, Cosson JF, Galan M, Hewison AJM (2009) Multiple paternity occurs with low frequency in the territorial roe deer, *Capreolus capreolus*. Biol J Linn Soc 97: 128-139.
163. Lacey EA, Wieczorek JR, Tucker PK (1997) Male mating behaviour and patterns of sperm precedence in Arctic ground squirrels. Anim Behav 53: 767-779.
164. Matsubayashi K, Mochizuki K (1982) Growth of male reproductive organs with observation of their seasonal morphologic changes in the Japanese monkey (*Macaca fuscata*). Jpn J Vet Sci 44: 891-902.
165. Runrong YE, Wenyang Z, Qinhua BAI, Jingxiao JIA (1990) Breeding of plateau pika under artificial conditions. Acta Therio Sinica 10: 287-293.
166. Yin B, Yang S, Wei W, Zhang Y (online early) Male reproductive success in plateau pikas (*Ochotona curzoniae*): A microsatellite analysis. Mamm Biol.
167. Bakker J, Baum MJ (2000) Neuroendocrine regulation of GnRH release in induced ovulators. Front Neuroendocrinol*.* 21: 220-262.
168. von Holst D, Hutzelmeyer H, Kaetzke P, Khaschei M, Schönheiter R (1999) Social rank, stress, fitness, and life expectancy in wild rabbits. Naturwissenschaften 86: 388-393.
169. Kays RW, Gittleman JL, Wayne RK (2000) Microsatellite analysis of kinkajou social organization. Mol Ecol 9: 743-751.
170. Roemer GW, Smith DA, Garcelon DK, Wayne RK (2001) The behavioural ecology of the island fox (Urocyon littoralis). J Zool 255: 1-14.
171. Ziwei L, Ruyong S, Jizeng D (1998) Seasonal reproductive cycles in male plateau pika (*Ochotona curzoniae*). Acta Therio Sinica 18: 42-49.
172. Vidya TNC, Balmforth Z, Le Roux A, Cherry MI (2009) Genetic structure, relatedness and helping behaviour in the yellow mongoose in a farmland and a natural habitat. J Zool 278: 57-64.
173. **Borkowska A, Borowski, Krysiuk K (2009)** Multiple paternity in free-living root voles (*Microtus oeconomus*). Behav Proc 825: 211-213.
174. Wiley RW (1992) Reproduction, postnatal development, and growth of the southern plains woodrat (*Neotoma micropus*) in western Texas. PhD, Texas Tech University, USA.
175. Dnate' Baxter B, Mendez-Harclerode FM, Fulhorst CF, Bradley RD (2009) A molecular examination of relatedness, multiple paternity, and cohabitation of the southern plains woodrat (Neotoma micropus). J Mammal 90: 819-831.
176. Poteaux C, Baubet E, Kaminski G, Brandt S, Dobson FS, Baudoin C (2009) Socio-genetic structure and mating system of a wild boar population. J Zool 278: 116-125.
177. Mauget R, Boissin J (1987) Seasonal changes in testis weight and testosterone concentration in the European wild boar (*Sus scrofa* L.). Anim Reprod Sci 13: 67-74.
178. Leader-Williams N, Rosser AM (1983) Ovarian characteristics and reproductive performance of reindeer, *Rangifer tarandus.*. J Reprod Fert 67: 247–256.
179. Røed KH, Holand Ø, Smith ME, Gjøstein H, Kumpula J, Nieminen M (2002) Reproductive success in reindeer males in a herd with varying sex ratio. Mol Ecol 11: 1239-1243.
